# Role of Infections in the Pathogenesis of Rheumatoid Arthritis: Focus on Mycobacteria

**DOI:** 10.3390/microorganisms8101459

**Published:** 2020-09-23

**Authors:** Marco Bo, Seyedesomaye Jasemi, Giuseppe Uras, Gian Luca Erre, Giuseppe Passiu, Leonardo A. Sechi

**Affiliations:** 1 Department of Biomedical Sciences, Section of Microbiology and Virology, University of Sassari, Viale San Pietro 43b, 07100 Sassari, Italy; 30039536@studenti.uniss.it (M.B.); somayejasemy@yahoo.com (S.J.); 2 Division of Molecular Therapeutics & Formulation, School of Pharmacy, The University of Nottingham, University Park Campus, Nottingham NG7 2RD, UK; Giuseppe.Uras@nottingham.ac.uk; 3 Department of Medical, Surgical and Experimental Sciences, Clinical and Experimental Medicine, University of Sassari, Viale San Pietro 43b, 07100 Sassari, Italy; gianluca.erre@aousassari.it (G.L.E.); passiu@uniss.it (G.P.); 4 Dipartimento di Specialità Mediche, Azienda Ospedaliero-Universitaria di Sassari, UOC of Rheumatology, Viale San Pietro 8, 07100 Sassari, Italy

**Keywords:** infections, mycobacteria, immune dysregulation, genes, rheumatoid arthritis

## Abstract

Rheumatoid arthritis (RA) is a systemic inflammatory autoimmune disease characterized by chronic erosive polyarthritis. A complex interaction between a favorable genetic background, and the presence of a specific immune response against a broad-spectrum of environmental factors seems to play a role in determining susceptibility to RA. Among different pathogens, *mycobacteria* (including *Mycobacterium avium* subspecies *paratuberculosis*, MAP), and *Epstein–Barr virus* (EBV), have extensively been proposed to promote specific cellular and humoral response in susceptible individuals, by activating pathways linked to RA development. In this review, we discuss the available experimental and clinical evidence on the interplay between mycobacterial and EBV infections, and the development of the immune dysregulation in RA.

## 1. Introduction

Rheumatoid arthritis (RA) is a chronic autoimmune disease, with a reported prevalence ranging between 0.5–1% worldwide [1,2,3]. RA is characterized by a systemic auto-immune disease, causing joint pain, swelling, and stiffness. RA usually affects hands, feet, and wrists, leading to progressive bone and cartilage damage, resulting in deformities, joints loss of function, and reduced independence in performing daily activities [4,5]. RA clinical manifestations are usually not confined to musculoskeletal system, but also involve cardiovascular system, kidneys, lungs, liver and skin [6]. RA onset is usually insidious. The most common scenario is characterized by symmetrical inflammatory involvement of small joints. However, in a non-negligible proportion of patients, no specific diagnosis can be made at the first presentation, due to atypical clinical manifestations and negativity to RA-specific biomarkers.

RA patients suffer from premature atherosclerosis and excessive cardiovascular disease burden. However, the prompt control of systemic inflammation due to the implementation of effective treatments in the early phases of disease, has led, in the past, to two decades of a significant reduction in the excess of cardiovascular disease burden. Moreover, the relative risk of death in the RA population is still significantly increased, compared to the general population, due to cancer and infections. It has been estimated that RA patients developing infectious complications may have a significant rise in death risk (up to 52%), with respect to the RA counterpart without history of infections [7].

The etiology of RA is complex and cannot be described solely by genetic factors and epigenetic mechanisms [8]. Environmental factors such as smoking, infections, and microbiota have been identified as risk factors to develop RA in susceptible individuals [8].

The role of infections in the development of autoimmune diseases has long been considered, since the infection with different pathogens can involve multiple pathways of the immune system, potentially triggering an autoimmune response [9].

The role of *Mycobacteria* in triggering and exacerbating the RA disease is noteworthy. Over the last two decades, a significant number of in vitro and in vivo studies have convincingly shown the presence of a biological (and likely pathogenic) link between the immune response against mycobacterial infections and the development of autoimmune diseases, including chronic inflammatory arthritis [10,11,12]. For instance, Liao et al. showed that RA patients are 2.28 and 6.24 times more likely to be infected by *Mycobacterium tuberculosis* (Mtb), and other *Mycobacterium* species, when compared to the general population [13]. Pathobiology of the disease itself, comorbid conditions, as well as the use of immunosuppressive treatment, may play a role as well [14,15].

The purpose of this paper is to review the available evidence about the link between mycobacterial, and other common infections, and immune dysregulation in RA.

## 2. RA Immunopathogenesis

RA is a chronic inflammatory disease characterized by synovial inflammation and bone damage, resulting from the proliferation of synovial fibroblasts, B and T lymphocytes, neutrophils, and monocytes [16,17,18].

A number of different genetic factors play a crucial role in RA pathogenesis, with RA heritability accounting for about 40–60% of RA susceptibility [19]. RA has extensively been associated to HLA-DR genes and non-HLA genes variants [20]. It has been reported that HLA alleles HLA-DRB1*01 and HLA-DRB1*04 are associated with RA susceptibility, with shared epitopes (SE) mechanisms [21]. Moreover, the HLA-DRB1*1001 allele triggers an immune response against citrullinated proteins [22].

A number of different studies have investigated the non-HLA genes’ role in RA pathogenesis and susceptibility, with the IL23A, PTPN22, and PAD14 genes being associated to RA [23,24,25,26,27].

HLA-DR genes have been linked to autoimmunity processes in RA, with the production of autoantibodies playing a central role, and with SE being the top risk factor, leading to anti-citrullinated peptide antibodies (anti-CCP) titers being significantly increased in the serum as well [19].

In particular, anti-CCP production has been linked to the induction of the autoimmune process in RA, highlighting the importance of HLA-DR genes involved in the SE mechanism.

The presence of immune cells in the synovial compartment is a typical hallmark of RA, triggered by a large number of different mechanisms, including cell–cell interactions, secretion of soluble mediators, autoantibodies, and signal transduction pathways of both innate and acquired immune response at various stages of the disease [28]. Macrophages, neutrophils, mast cells, and natural killer (NK) cells are involved in the development of inflammatory response in the joint, as a result of innate immune response activation. Antigen-presenting cells (APCs), such as macrophages, and effector cells, promote inflammation and mediate bone and cartilage destruction by releasing pro-inflammatory factors, such as tumor necrosis factor alpha (TNF-α), interleukin-1B (IL-1B), IL-6, IL-18, IL-23, reactive oxygen species (ROS), and matrix-degrading enzymes [29,30].

In particular, TNF-α plays a central role in the pathogenesis of the disease by increasing inflammatory cytokines levels, activating macrophages and lymphocytes. For these reasons, TNF-α has been extensively identified as a therapeutic target, leading to the development of several TNF inhibitors that actually represent the mainstay of treatment of moderate-severe disease modifying anti-rheumatic drugs (DMARDs) refractory RA [31].

As previously mentioned, neutrophils play an important role in the RA pathogenesis, accounting for 80–90% of the synovial fluid cells in RA patients [28,29,30]. These cells exacerbate inflammation and tissue destruction by releasing pro-inflammatory cytokines, ROS, granules containing destructive enzymes, and the formation of neutrophil extracellular trap (NET) [8,32].

In addition, Toll-like receptors (TLRs) signaling pathways play an important role in the pathogenesis of RA [33,34]. TLRs receptors are divided into two main categories, extracellular receptors (TLR-1, 2, 4, 5, 6) and intracellular receptors (TLR-3, 5, 7), which interact with components of the bacterial cell surface and ligands found in the endosomal compartment, respectively. As a result of TLRs activation, pro-inflammatory cytokines and chemokines, such as TNFα and IFNα/β, are expressed via MyD88-dependent or independent pathways activation [33].

In RA patients, the chronic inflammatory processes that characterize the patients’ joints may be triggered by TLRs aberrant activation. In particular, it was found that, in RA patients, TLRs are increased in both peripheral blood monocytes (TLR-2 and 4), synovial fibroblast (TLR-3 and 7), and in synovial fluid macrophages (TLR-2 and 4) [35,36]. Moreover, microbial and endogenous ligands were reported to be able to activate TLRs in patients’ derived cells. In particular, bacterial LPS and peptidoglycan induced the expression of IL-6 and CXCL8, via TLR-2 binding, in RA synovial fibroblast. Moreover, macrophages with an increased expression of TLR-2 resulted in an aberrant response to bacterial peptidoglycan [36]. The same results were observed in RA patients, with upregulated response to TLR-2 and TLR-4 ligands, in peripheral blood monocytes and synovial macrophages [37]. Other than that, endogenous ligands, for instance the stress response protein gp96, could potentially result in an incorrect activation of TLRs pathway [33]. Cell culture studies with TLR-3, 7, 8, and 9 inhibitors resulted in reduced levels of inflammatory cytokines (TNFα and IL-6), while agonists significantly increased the secretion of such molecules, suggesting that a viral infection or an endogenous ligand can potentially trigger the chronic inflammation in RA patients [38]. Further to this, non-apoptotic Fas-FasL signaling, which regulates the activation threshold for macrophages and fibroblast in the synovial fluid, may be aberrant in RA patients, resulting in an increased sensitivity to TLRs activation and, therefore, a chronic inflammation [33]. Thus, upregulated expression, the presence of microbial and endogenous ligands, and increased sensitivity to TLRs signaling may confer a crucial role to TLRs in RA pathogenesis.

Despite RA being a type 1 T helper (Th1)-mediated disease, recent available evidence suggests that T helper 17 cells (Th17) are an important effector cell population in RA pathogenesis as well [39,40,41]. The secretion of IL-17A cytokines, by Th17 cells, activates a number of pathways such as Fibroblast-like synoviocytes (FLS), maturation and function of osteoclasts, activation of neutrophils, macrophages and B cells [40,41]. Th17 cells play an important role in RA pathogenesis, by synthesizing other cytokines and chemokines, including IL-17F, IL-22, INF-Y, TNF-*α*, granulocyte macrophage colony-stimulating factor (GM-CSF), and chemokine (C-C motif) ligand 20 (CCL20) [42].

Further to this, humoral immunity has been highlighted as a potential factor in RA etiopathogenesis. For instance, the presence of anti-IgG FC autoantibodies was reported in 70–80% of RA patients, as well as anti-CCP [43]. Despite the role of these auto-antibodies being still unclear, multiple studies have shown that seropositive patients are likely to develop a more severe disease when compared to seronegative patients [44,45]. Serum rheumatoid factor (RF) and anti-CCP are currently used as biomarkers for the diagnosis of RA [6]. These antibodies (Abs) appear many years prior to RA onset, during the so-called “pre-clinical” course of the disease [46,47]. This finding supports the hypothesis that early steps of RA pathogenesis may originate in an extra-articular environment, such as the mucosal interface of gastrointestinal tract and respiratory system [48,49,50,51].

To date, different molecular mechanisms have been reported to play a role in autoimmune processes, such as pathogen/host interaction, and molecular mimicry [52,53]. Moreover, it has also been shown that cross-reactive Abs produced in the context of microbial infections have the potential to cause damage to host tissues [54,55].

In the presence of unfavorable conditions, the host’s immune response to pathogens, as well as the pathogen’s direct attack against the host, may lead to self-tissue damage and release of auto-antigen, resulting in the development of a self-specific immune response mounted to the host tissue [56,57].

In addition, bacterial infections can lead to the proliferation, and differentiation, of B and T lymphocytes, without their antigenic specificity, resulting in direct inflammatory responses against the host, triggering the polyclonal lymphocyte activation [58]. Other than that, microbial infection may trigger inflammatory pathways, by activating reactive lymphocyte cells, leading to autoimmune responses, called bystander activation [58].

Different studies have demonstrated that molecular mimicry between *Epstein–Barr virus* (EBV), *Mycobacterium avium* subspecies *paratuberculosis* (MAP) and host peptides acts as an RA pathogenic mechanism. MAP and EBV infections can lead to the deregulation of the Interferon regulatory factor 5 (IRF5) pathway. The frequency of Abs reactivity against IRF5 was increased in RA patients compared to healthy controls (56% vs. 9%, *p* < 0.0001) [59]. A similar trend was found for Abs against the EBV tegument protein called BOLF1, where the frequency of reactivity was 44% vs. 9% (*p* < 0.0001), in RA patients and healthy controls, respectively [59]. Finally, it was found that Abs against MAP_4027 have a higher reactivity in RA patients compared to the control group (21% vs. 9%, *p* < 0.0076) [59].

Experiments with antigen-induced arthritis performed in IRF5 conditional KO mice strengthened the hypothesis that Abs generated against the three homologues peptides are cross-reactive. This discovery supports the hypothesis that IRF5 is a potential auto-immune target of RA. However, further studies in a larger group of patients are needed to further confirm these findings [60].

## 3. The Role of Mycobacterial Infections in Rheumatoid Arthritis

*Mycobacterium* genus has more than 170 species, most of which are environmental organisms [61]. Mycobacterial infections include tuberculosis and non-tuberculous mycobacterial infections, which cause subacute clinical symptoms with granulomatous inflammation [62].

Different studies showed the link between the immune response to mycobacterial infections and autoimmune diseases, especially autoimmune arthritis [10,11,12,63,64,65,66]. Poncet et al. presented the first study on this association in the late 19th century, after reporting that a type of aseptic polyarthritis was developed in patients with active tuberculosis, later named Poncet’s disease [67]. In addition, this association was strengthened after the observation of a seronegative form of oligoarthritis following immunotherapy with Bacillus Calmette–Guerin (BCG) vaccine [68]. Various studies have shown the presence of mycobacterium antigens in RA patients’ joints [69,70,71,72]. Moreover, increased levels of Abs against *Mycobacterium* in the serum [73,74,75] and the presence of active T cells in the synovium have been reported in RA patients [76,77]. In a collagen-induced arthritis (CIA) mice model, mice treated with collagen plus killed Mtb developed severe arthritis, while, on the contrary, mice treated with collagen emulsion alone did not develop arthritis [78].

In CIA, arthritis is normally induced by immunization with autologous or heterologous type II collagen in adjuvant. Susceptibility to collagen-induced arthritis is strongly associated with major histocompatibility complex class II genes, and the immune-pathogenesis of CIA involved both a T-cell and B-cell specific response to type II collagen [79]. The pathological features of CIA include a proliferative synovitis with the infiltration of polymorphonuclear and mononuclear cells, pannus formation, cartilage degradation, erosion of bone, and fibrosis. As in RA, pro-inflammatory cytokines, such as tumor necrosis factor α (TNFα) and interleukin (IL)-1β, are abundantly expressed in the arthritic joints of mice with CIA, and the blockade of these molecules results in a reduction of disease severity [79].

**Figure 1:**
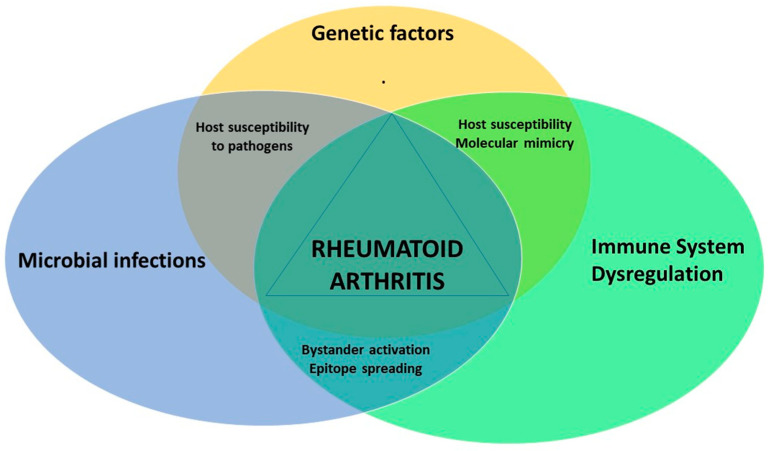
Interaction between different factors in driving rheumatoid arthritis (RA) pathogenesis.

*Mycobacterium* is a potent immunogen, often causing uncontrolled immune responses that are likely to play a role in RA pathogenesis [80]. Complete Freund’s adjuvant (CFA), which contains inactivated *Mycobacteria*, is used as immunopotentiator (booster), to develop several animal models of autoimmune diseases, such as adjuvant-induced arthritis [81]. Moreover, studies have shown that components of *Mycobacteria*, such as muramyl dipeptide, glycolipids, and lipoarabinomannan (LAM) are all capable of replacing *Mycobacteria* in CFA for immunity induction [81] (Figure 1).

Other studies have shown that in RA patients some mycobacterial lipids, named pathogen associated molecular patterns (PAMPs), are able to increase the immune response via TLR-2 and TLR-4 binding, resulting in the increased maturation of dendritic cells, ROS production, synthesis of pro-inflammatory RA cytokines (such as IL-1, IL-6, IL-17 and IL-23), and TNF-α secretion by neutrophils [82].

Gene expression analysis, and subsequent gene ontology study, revealed that genes belonging to T cell receptor signaling pathway, TLR signaling pathway, and virus defense signaling pathway, such as TLR-5, TNFSF10/TRAIL (tumor necrosis factor (ligand) family, member 10/TNF-related apoptosis-inducing ligand), PPP1R1613/TIMAP (protein phosphatase 1 regulatory subunit 16B), SIAH1 (E3 ubiquitin protein ligase 1), PIK3IP1 (phosphoinositide-3-kinase protein 1) and IL-17 are significantly dysregulated in TBC and RA patients [83].

Molecular mimicry between mycobacterial antigens and host proteins is one of the possible explanations regarding the role of immune response against *Mycobacteria* in the development of autoimmune diseases such as RA [84,85]. Supporting this preposition, immunization against *M. tuberculosis* has been reported to cause arthritis due to cross reaction with cartilage proteoglycans [86]. In addition, some studies have shown a non-negligible prevalence of anti-CCP and anti-arginine-containing peptide (anti-CAP) positivity in the serum of patients with mycobacterial infections [87,88,89,90]. Moreover, polyclonal antibodies against human lactoferrin, cross-reacted against *mycobacterial* antigens, further support the role of such molecules in triggering molecular mimicry mechanisms in RA [86,91]. There is ample evidence suggesting that TB-reactive T cells can be potentially arthritogenic, as they react, specifically, against both cartilage and *Mtb* antigens [65,92]. In a case-control study, Bo et al. found increased levels of Abs against two main proteins of MAP, named protein tyrosine phosphatase A (PtpA) and protein kinase G (PknG), in RA patients compared to healthy controls. This finding of a previous exposure of RA patients to MAP infection suggests a potential role of MAP infection in the RA pathogenesis [75].

It is important to highlight that a number of mycobacterium antigens, such as Mtb, are associated with autoimmune diseases, such as autoimmune arthritis, sarcoidosis, systemic lupus erythematosus [65,86,91,93,94,95,96,97,98], where the most prevalent mycobacterial antigen detected was the heat shock protein 65 (HSP65) [65,86,91,93,94]. The latter is an immunodominant protein similar to several human proteins, such as lactoferrin transferrin, alphaB-crystallin in terms of sequence and conformation [86,91,95]. HSP65 region between aa180–188 can stimulate auto-reactive T lymphocytes that react with cartilage-resident self-proteins [99]. HSP65 increases the responses of mononuclear cells in the synovial fluid of RA patients, and the clonal expansion of T cells against mycobacterium HSP65 was detected in RA patients’ blood and synovial fluid [82,83]. Mycobacteria Heat shock protein 16 (HSP16), 70 (HSP70) and HSP65 demonstrated 18–60% identity to their human homologues [95,100,101]. Autoimmune response to Mycobacterial (Myc) HSP70 and human binding immunoglobulin protein (Bip), a member of the human HSP70 family, has also been reported in RA patients [102]. Shoda’s study showed that the similarity between Myc HSP70 _287–306_ and human Bip_336–355_ epitopes can lead to a broken immune tolerance, triggering an auto-immune response as a result of the T cells’ inability to distinguish between self- and pathogens’ antigens [103] (Figure 1).

A different approach to investigate the similarities between the virulence factors of Mtb and human proteins are bioinformatics models. HLA class I and II restricted T cell epitopes from host proteins that share bacterial and homologues human HSP60 specialty KPLVIIAEDVDGEALSTLVLN, bind to many HLA class I and class II alleles, including HLA-DRB1: *01:01, *03:01, *04:01, *07:01*, 08:02, *11:01, *13:01, *15:01, A*01:01, A*02:01, A*03:01, A*011:01, A*024:02, A*07:02, A*08:01 [104].

Findings indicated the presence of matching 22 B-cell, 79 human leucocyte antigen (HLA) class II and 16 HLA class I specific predicted epitopes in these virulence factors having human homologs [105]. In addition, in silico analysis showed that T cell cross-reactive epitopes between *M. tuberculosis* and the human proteome can be considered as vaccine candidates [106,107,108].

Genomic analysis showed that single nucleotide polymorphism (SNPs) in immune related genes play a role in increasing the severity of mycobacterial disease, and its association with autoimmune diseases [109,110,111,112]. One of the most common genes studied in this context is the *SLC11A1* gene (solute carrier family 11 member a1). The protein encoded by the *SLC11A1* gene, named natural resistance associated macrophage protein 1 (NRAMP1), plays an important role, activating macrophages and the innate immune system [113]. The expression of NRAMP1 causes acidification of the phagosome, eventually leading to the destruction of the intracellular pathogen, whilst mutations in *SLC11A1* gene cause intracellular pathogens survival [114]. According to several mutational screenings, mutations in *SLC11A1* are linked to autoimmune diseases, such as RA [109,115], multiple sclerosis [116,117], inflammatory bowel disease [110,118], and type 1 diabetes mellitus [111,119]. Examination of SNPs in *TNF-α* gene, and its receptors (*TNFRSF1A/TNFRSF1B*), in RA patients compared to HCs, reported that some SNPs, TNFRSF1A:rs767455 and TNFRSF1B:rs3397, are linked to TNFRSF1B downregulation, increased susceptibility to MAP infection, increased inflammation and osteocalcin deficiency, and, possibly, increased osteoporosis [120]. Sharp et al. showed that the SNPs in *PTPN2/22* genes (protein tyrosine phosphatase non-receptor type 2 and 22) are linked to increased sensitivity to MAP infection and, therefore, increased T lymphocyte response, and IFN-γ expression in RA patients [112] (Figure 1, Table 1).

Furthermore, infection is one of the most important complications in RA patients [121]. The risk of infectious diseases increases in the RA, due to immunological dysfunction, immunosuppressive therapy, and associated comorbidities [122]. The activation of latent tuberculosis is a major concern during the treatment of RA patients. The risk of tuberculosis, as well as the risk of *non-tuberculous Mycobacteria*, increases from about 1.6 to 25 times during the treatment with TNF blockers [123,124]. In addition, there are reports of some other species of Mycobacterium associated with RA disease that make it difficult to treat the disease. There have been case reports of other Mycobacterium species in infections and arthritis and other parts of the body in RA patients around the world [125,126,127,128,129,130,131,132]. Furthermore, the risk of death in RA patients with mycobacterial infection was higher than that in patients without infection [13].

The breakdown of different mechanisms ultimately leads to the activation of molecular mimicry, bystander activation, and epitope spreading. Triggering such mechanisms, along with the presence of either microbial infections, genetic variants, and immune system dysregulation, results in the development of RA disease.

## 4. Other Infections Associated with RA

The evidence of an association between microorganism’s infection and RA disease dates back to the 1870s, with suspected pathogens still being added to this list [133]. Using different laboratory methods, have allowed the detection in RA patients’ joints, and serum, of several microorganisms, or their components, such as *Porphyromonas gingivalis* [134,135], *Mycoplasma* [136,137,138], *Bordetella* [139,140], *Haemophilus* [139,140], *Acinetobacter* [140], Parvovirus [141,142], Epstein–Barr virus (EBV) [143,144,145,146,147] and Cytomegalovirus (CMV) [144] (Table 1).

Immune responses against microbes, such as *Porphyromonas gingivalis* [148,149,150], EBV [151,152,153,154,155,156], Human T-Lymphotropic Virus (HTLV) [157], *Mycoplasma* [158,159], Parvovirus B19 [160], Papilloma Virus [161], and Endogenous retroviruses (HERV) [162], have been reported in RA patients. On the other hand, some animal models of arthritis were developed, exploiting the infection of some pathogens, such as *P. gingivalis* [163,164,165,166], *Mycoplasma* [167], and EBV [168,169] [Table 1].

Laboratory and clinical studies have shown that *Porphyromonas gingivalis* is the most common microorganism associated with RA etiopathology [170]. Of note, it has been reported a similarity of up to 82% between *P. gingivalis* enolase and human α-enolase within the 17-amino acid immunodominant region, and Abs levels against bacterial enolase were related to the levels of Abs against the human enolase [171,172,173].

Reports of both microbial components, and the immune response against them, in RA patients’ joints tissue and serum were exploited to develop mice models of RA, through the injection of different microorganisms in the mouse joints.

Another microorganism associated to RA is EBV. Again, a cross-reaction mechanism was detected between anti-p107 EBV Abs and human denatured collagen and creatine. This molecular mimicry mechanism may increase autoreactive T cell activation and proliferation [173]. As previously mentioned, Abs against EBV and MAP antigen BOLF1, MAP_4027_18–32_ human homologous (IRF5 epitope) were significantly higher in RA patients than healthy controls, indicating that these microorganisms may be involved in RA pathogenesis, with the production of cross-reactive Abs being a central mechanism to trigger autoimmune disease [174].

*Proteus mirabilis* is another RA-related pathogen [175,176]. Studies by serological and proteomics methods have shown that there are similarities between hemolysin (ESRRAL) and urease (IRRE7) sequences in *P. mirabilis*, with HLA-DR (EQRRAA) and collagen XI (LRREI) antigen epitopes [177,178,179]. In addition, cross-reactivity is present between bacterial hemolysin and urease enzymes with human proteome, which, subsequently, activates B lymphocytes and stimulates the production of autoantibodies. Moreover, Abs against ESRRAL and EQRRAA have been detected in RA patients [177,179].

Another RA-related pathogen is *Escherichia coli*. The QKRAA motif of the dnaJ class of heat-shock proteins from *E. coli* is as well present in HLA-DRB1 antigens. QKRAA motif strongly activates the T cells in the synovial region in RA patients. This activation may result in a cross reaction against the host dnaJ heat-shock proteins that are expressed in the synovial microenvironment [23,180].

Another non-specific inflammatory response, named bystander activation, can be exploited by microorganisms to exacerbate RA. In vitro and in vivo studies have shown that bacterial lipopolysaccharides stimulate osteoclast formation and bone resorption through TLRs pathway activation [181,182,183]. Lipopolysaccharide (LPS) can stimulate macrophages to secrete pre-inflammatory cytokines [184,185]. *Porphyromonas* LPS stimulated the activation of monocytes and the production of RA-related cytokines, such as IL-1 and IL-23, via TLR pathway [186,187], ultimately promoting osteoclast formation and the bone resorption [188,189]. Concomitant injection of LPS with moramil dipeptide (MDP) also increases the expression of pro-inflammatory cytokines by monocyte cell culture [190,191]. *Mycoplasma* Glycolipid antigens (GGPL-III) significantly increased the production of TNF-α and IL-6 in the peripheral blood and the proliferation of synovial fibroblasts [136].

The EBV DNA increased the secretion of pro-inflammatory cytokines, such as IL-17, IL-23 and TNF-α in mice, which could lead to, or exacerbate, autoimmune diseases [192]. In addition, EBV and *E.coli* DNA ligation to endosomal TLR-9 leads to increased IL-17A expression, which is an essential cytokine in the synovial environment [193]. The EBV infection in human lymphocytes under in vitro conditions could cause the expansion of non-specific B lymphocytes and TCD8^+^ cells, leading to the production of polyclonal antibodies and the activation of cytotoxic T lymphocytes [151,152,194]. Accordingly, T lymphocyte response to EBV [153,154] and CMV [195] has been reported in inflamed joints of RA patients.

Generation of neo-antigen and epitope spreading is another mechanism triggered by microorganism infections involved in RA pathogenesis. For instance, *Porphyromonas gingivalis* is the only bacterium that produces the peptidylarginine deiminase (PAD)-enzyme with citrullination activity. Host proteins post-translation modifications are catalyzed by this enzyme, resulting in the production of new antigens. It has also been reported that *P. gingivalis,* through PAD-enzyme activity, is able to generate neo-antigens in the joint, including citrullinated-fibrinogen, α-enolase and vimentin, resulting in the stimulation of the auto-immune response [196,197,198].

By producing proteinase enzymes, *Porphyromonas* increases apoptosis in chondrocyte cells, thereby destroying cartilage tissue and deforming the joint, which is an important mechanism in RA pathogenesis [170,199].

## 5. Conclusions

There is ample evidence showing a link between different microbial pathogens and RA development and progression. On the other hand, favorable genetic background, different environmental factors, including lifestyle and immunosuppressive treatment, are also involved in the increased risk of infection in various stages of RA, from the pre-clinical phase to the established/late phase. A better comprehension of the intricate relationship between microbial pathogens and RA may help in the future to develop effective strategies to block early pathogenic steps of disease, thus preventing the development of the clinical phase of RA.

## Figures and Tables

**Table 1:** In vivo and in vitro studies of microorganisms related to RA.

**Presence of microbial contents in RA patients tissues and serum**	*Mycobacteria*, *P. gingivalis*, EBV, *Mycoplasma*, *Bordetalla*, *Haemophilus*, *Acinetobacter*, *Parvovirus*, CMV, Bacterial cell wall
**Presence of immune response to infection in RA patients tissues and serum**	*Mycobacteria*, *P. gingivalis*, EBV, HTLV, *Mycoplasma*, *Parvovirus B19*, *Papilloma virus*, HERV
**Induction of Arthritis by Infections in Animal Models** 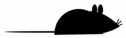	*Mycobacteria*, *P. gingivalis*, *Mycoplasma*, *EBV*

## References

[1] Simon T.A., Kawabata H., Ray N., Baheti A., Suissa S., Esdaile J.M. (2017). Prevalence of co-existing autoimmune disease in rheumatoid arthritis: A cross-sectional study. Adv. Ther..

[2] Alivernini S., Tolusso B., Petricca L., Ferraccioli G., Gremese E. (2019). Chapter 46—Rheumatoid arthritis. Mosaic of Autoimmunity.

[3] de Brito Rocha S., Baldo D.C., Andrade L.E.C. (2019). Clinical and pathophysiologic relevance of autoantibodies in rheumatoid arthritis. Adv. Rheumatol..

[4] Huizinga T.W., Pincus T. (2010). Rheumatoid arthritis. Ann. Intern. Med..

[5] Grassi W., De Angelis R., Lamanna G., Cervini C. (1998). The clinical featuRes. of rheumatoid arthritis. Eur. J. Radiol..

[6] van Delft M.A.M., Huizinga T.W.J. (2020). An overview of autoantibodies in rheumatoid arthritis. J. Autoimmun..

[7] Ma X., Xu S. (2013). TNF inhibitor therapy for rheumatoid arthritis. Biomed. Rep..

[8] Croia C., Bursi R., Sutera D., Petrelli F., Alunno A., Puxeddu I. (2019). One year in review 2019: Pathogenesis of rheumatoid arthritis. Clin. Exp. Rheumatol..

[9] Hussein H.M., Rahal E.A. (2019). The role of viral infections in the development of autoimmune diseases. Crit Rev. Microbiol..

[10] Atkin S.L., Welbury R.R., Stanfield E., Beavis D., Iwais B., Dick W.C. (1987). Clinical and laboratory studies of inflammatory polyarthritis in patients with leprosy in Papua New Guinea. Ann. Rheum. Dis..

[11] Rook G.A. (1988). Rheumatoid arthritis, mycobacterial antigens and agalactosyl IgG. Scand. J. Immunol..

[12] Shoenfeld Y., Isenberg D.A. (1988). Mycobacteria and autoimmunity. Immunol. Today.

[13] Liao T.L., Lin C.H., Shen G.H., Chang C.L., Lin C.F., Chen D.Y. (2015). Risk for mycobacterial disease among patients with rheumatoid arthritis, Taiwan, 2001–2011. Emerg. Infect. Dis..

[14] Listing J., Gerhold K., Zink A. (2012). The risk of infections associated with rheumatoid arthritis, with its comorbidity and treatment. Rheumatology.

[15] Mehta B., Pedro S., Ozen G., Kalil A., Wolfe F., Mikuls T., Michaud K. (2019). Serious infection risk in rheumatoid arthritis compared with non-inflammatory rheumatic and musculoskeletal diseases: A US national cohort study. RMD Open.

[16] McInnes I.B., Schett G. (2007). Cytokines in the pathogenesis of rheumatoid arthritis. Nat. Rev. Immunol..

[17] Brennan F.M., McInnes I.B. (2008). Evidence that cytokines play a role in rheumatoid arthritis. J. Clin. Investig..

[18] Coutant F., Miossec P. (2020). Evolving concepts of the pathogenesis of rheumatoid arthritis with focus on the early and late stages. Curr. Opin. Rheumatol..

[19] Karami J., Aslani S., Jamshidi A., Garshasbi M., Mahmoudi M. (2019). Genetic implications in the pathogenesis of rheumatoid arthritis; an updated review. Gene.

[20] Lee J.C., Espéli M., Anderson C.A., Linterman M.A., Pocock J.M., Williams N.J., Roberts R., Viatte S., Fu B., Peshu N. (2013). Human SNP links differential outcomes in inflammatory and infectious disease to a FOXO3-regulated pathway. Cell.

[21] Gregersen P.K., Silver J., Winchester R.J. (1987). The shared epitope hypothesis. an approach to understanding the molecular genetics of susceptibility to rheumatoid arthritis. Arthritis Rheum..

[22] van der Helm-van Mil A.H.M., Verpoort K.N., le Cessie S., Huizinga T.W.J., de Vries R.R.P., Toes R.E.M. (2007). The HLA–DRB1 shared epitope alleles differ in the interaction with smoking and predisposition to antibodies to cyclic citrullinated peptide. Arthritis Rheum..

[23] Albani S., Keystone E.C., Nelson J.L., Ollier W.E., La Cava A., Montemayor A.C., Weber D.A., Montecucco C., Martini A., Carson D.A. (1995). Positive selection in autoimmunity: Abnormal immune responses to a bacterial dnaJ antigenic determinant in patients with early rheumatoid arthritis. Nat. Med..

[24] Bax M., van Heemst J., Huizinga T.W.J., Toes R.E.M. (2011). Genetics of rheumatoid arthritis: What have we learned?. Immunogenetics.

[25] Cha S., Choi C.-B., Han T.-U., Kang C.P., Kang C., Bae S.-C. (2007). Association of Anti–Cyclic citrullinated peptide antibody levels with PADI4 haplotypes in early rheumatoid arthritis and with shared epitope alleles in very late rheumatoid arthritis. Arthritis Rheum..

[26] Faragó B., Magyari L., Sáfrány E., Csöngei V., Járomi L., Horvatovich K., Sipeky C., Maász A., Radics J., Gyetvai Á. (2008). Functional variants of interleukin-23 receptor gene confer risk for rheumatoid arthritis but not for systemic sclerosis. Ann. Rheum. Dis..

[27] Farago B., Talian G.C., Komlosi K., Nagy G., Berki T., Gyetvai A., Szekanecz Z., Nyarady Z., Kiss C.G., Nemeth P. (2009). Protein tyrosine phosphatase gene C1858T allele confers risk for rheumatoid arthritis in Hungarian subjects. Rheumatol. Int..

[28] Alivernini S., Tolusso B., Petricca L., Ferraccioli G., Gremese E. (2019). Chapter 16—Rheumatoid arthritis. Mosaic of Autoimmunity.

[29] Klareskog L., Catrina A.I., Paget S. (2009). Rheumatoid arthritis. Lancet.

[30] McInnes I.B., Schett G. (2011). The pathogenesis of rheumatoid arthritis. N. Engl. J. Med..

[31] Farrugia M., Baron B. (2016). The role of TNF-α in rheumatoid arthritis: A focus on regulatory T cells. J. Clin. Transl. Res..

[32] Calabresi E., Petrelli F., Bonifacio A.F., Puxeddu I., Alunno A. (2018). One year in review 2018: Pathogenesis of rheumatoid arthritis. Clin. Exp. Rheumatol..

[33] Huang Q.Q., Pope R.M. (2009). The role of toll-like receptors in rheumatoid arthritis. Curr. Rheumatol. Rep..

[34] Elshabrawy H.A., Essani A.E., Szekanecz Z., Fox D.A., Shahrara S. (2017). TLRs, future potential therapeutic targets for RA. AutoImmun. Rev..

[35] Ospelt C., Brentano F., Rengel Y., Stanczyk J., Kolling C., Tak P.P., Gay R.E., Gay S., Kyburz D. (2008). Overexpression of toll-like receptors 3 and 4 in synovial tissue from patients with early rheumatoid arthritis: Toll-like receptor expression in early and longstanding arthritis. Arthritis Rheum..

[36] Huang Q., Ma Y., Adebayo A., Pope R.M. (2007). Increased macrophage activation mediated through toll-like receptors in rheumatoid arthritis. Arthritis Rheum..

[37] Kowalski M.L., Wolska A., Grzegorczyk J., Hilt J., Jarzebska M., Drobniewski M., Synder M., Kurowski M. (2008). Increased responsiveness to toll-like receptor 4 stimulation in peripheral blood mononuclear cells from patients with recent onset rheumatoid arthritis. Mediat. Inflamm..

[38] Sacre S.M., Lo A., Gregory B., Simmonds R.E., Williams L., Feldmann M., Brennan F.M., Foxwell B.M. (2008). Inhibitors of TLR8 reduce TNF production from human rheumatoid synovial membrane cultures. J. Immunol..

[39] Doorenspleet M.E., Klarenbeek P.L., de Hair M.J., van Schaik B.D., Esveldt R.E., van Kampen A.H., Gerlag D.M., Musters A., Baas F., Tak P.P. (2014). Rheumatoid arthritis synovial tissue harbours dominant B-cell and plasma-cell clones associated with autoreactivity. Ann. Rheum. Dis..

[40] Lubberts E. (2015). The IL-23-IL-17 axis in inflammatory arthritis. Nat. Rev. Rheumatol..

[41] Gaffen S.L., Jain R., Garg A.V., Cua D.J. (2014). The IL-23-IL-17 immune axis: From mechanisms to therapeutic testing. Nat. Rev. Immunol..

[42] Paulissen S.M., van Hamburg J.P., Dankers W., Lubberts E. (2015). The role and modulation of CCR6+ Th17 cell populations in rheumatoid arthritis. Cytokine.

[43] Andersson A.K., Li C., Brennan F.M. (2008). Recent developments in the immunobiology of rheumatoid arthritis. Arthritis Res. Ther..

[44] Anaya J.M., Shoenfeld Y., Rojas-Villarraga A., Levy R.A., Cervera R. (2013). Autoimmunity: From Bench to Bedside.

[45] Abbas A.K.L.A., Pillai S. (2012). Cellular and Molecularimmunology.

[46] Demoruelle M.K., Deane K.D., Holers V.M. (2014). When and where does inflammation begin in rheumatoid arthritis?. Curr. Opin. Rheumatol..

[47] Deane K.D., Demoruelle M.K., Kelmenson L.B., Kuhn K.A., Norris J.M., Holers V.M. (2017). Genetic and environmental risk factors for rheumatoid arthritis. Best Pract. Res. Clin. Rheumatol..

[48] Klareskog L., Stolt P., Lundberg K., Källberg H., Bengtsson C., Grunewald J., Rönnelid J., Harris H.E., Ulfgren A.K., Rantapää-Dahlqvist S. (2006). A new model for an etiology of rheumatoid arthritis: Smoking may trigger HLA-DR (shared epitope)-restricted immune reactions to autoantigens modified by citrullination. Arthritis Rheum..

[49] Rosenstein E.D., Greenwald R.A., Kushner L.J., Weissmann G. (2004). Hypothesis: The humoral immune response to oral bacteria provides a stimulus for the development of rheumatoid arthritis. Inflammation.

[50] Malmström V., Catrina A.I., Klareskog L. (2017). The immunopathogenesis of seropositive rheumatoid arthritis: From triggering to targeting. Nat. Rev. Immunol..

[51] Gizinski A.M., Mascolo M., Loucks J.L., Kervitsky A., Meehan R.T., Brown K.K., Holers V.M., Deane K.D. (2009). Rheumatoid arthritis (RA)-specific autoantibodies in patients with interstitial lung disease and absence of clinically apparent articular RA. Clin. Rheumatol..

[52] Oldstone M.B. (1987). Molecular mimicry and autoimmune disease. Cell.

[53] Venigalla S.S.K., Premakumar S., Janakiraman V. (2020). A possible role for autoimmunity through molecular mimicry in alphavirus mediated arthritis. Sci. Rep..

[54] Root-Bernstein R., Fairweather D. (2014). Complexities in the relationship between infection and autoimmunity. Curr. Allergy Asthma Rep..

[55] Thaper D., Prabha V. (2018). Molecular mimicry: An explanation for autoimmune diseases and infertility. Scand. J. Immunol..

[56] Arleevskaya M.I., Kravtsova O.A., Lemerle J., Renaudineau Y., Tsibulkin A.P. (2016). How rheumatoid arthritis can result from provocation of the immune system by microorganisms and viruses. Front. Microbiol..

[57] Ercolini A.M., Miller S.D. (2009). The role of infections in autoimmune disease. Clin. Exp. Immunol..

[58] Fujinami R.S., von Herrath M.G., Christen U., Whitton J.L. (2006). Molecular mimicry, bystander activation, or viral persistence: Infections and autoimmune disease. Clin. Microbiol. Rev..

[59] Bo M., Niegowska M., Erre G.L., Piras M., Longu M.G., Manchia P., Manca M., Passiu G., Sechi L.A. (2018). Rheumatoid arthritis patient antibodies highly recognize IL-2 in the immune response pathway involving IRF5 and EBV antigens. Sci. Rep..

[60] Bo M., Niegowska M., Eames H.L., Almuttaqi H., Arru G., Erre G.L., Passiu G., Khoyratty T.E., van Grinsven E., Udalova I.A. (2020). Antibody response to homologous epitopes of Epstein-Barr virus, Mycobacterium avium subsp. paratuberculosis and IRF5 in patients with different connective tissue diseases and in mouse model of antigen-induced arthritis. J. Transl. Autoimmun..

[61] Gagneux S. (2018). Ecology and evolution of *Mycobacterium tuberculosis*. Nat. Rev. Microbiol..

[62] Cronan M.R., Beerman R.W., Rosenberg A.F., Saelens J.W., Johnson M.G., Oehlers S.H., Sisk D.M., Jurcic Smith K.L., Medvitz N.A., Miller S.E. (2016). Macrophage epithelial reprogramming underlies mycobacterial granuloma formation and promotes infection. Immunity.

[63] Birnbaum G., Kotilinek L., Albrecht L. (1993). Spinal fluid lymphocytes from a subgroup of multiple sclerosis patients respond to mycobacterial antigens. Ann. Neurol..

[64] Mor F., Cohen I.R. (1992). T cells in the lesion of experimental autoimmune encephalomyelitis. Enrichment for reactivities to myelin basic protein and to heat shock proteins. J. Clin. Investig..

[65] Res P.C., Schaar C.G., Breedveld F.C., van Eden W., van Embden J.D., Cohen I.R., de Vries R.R. (1988). Synovial fluid T cell reactivity against 65 kD heat shock protein of mycobacteria in early chronic arthritis. Lancet.

[66] Salvetti M., Ristori G., Buttinelli C., Fiori P., Falcone M., Britton W., Adams E., Paone G., Grasso M.G., Pozzilli C. (1996). The immune response to mycobacterial 70-kDa heat shock proteins frequently involves autoreactive T cells and is quantitatively disregulated in multiple sclerosis. J. NeuroImmunol..

[67] Poncet A. (1897). De la polyarthrite tuberculeuse deformante oupseudorheumatisme chronique tuberculeux. Congr. Fr. Chir..

[68] Torisu M., Miyahara T., Shinohara N., Ohsato K., Sonozaki H. (1978). A new side effect of BCG immunotherapy —BCG-induced arthritis in man. Cancer Immunol. Immunother..

[69] Kempsell K.E., Cox C.J., McColm A.A., Bagshaw J.A., Reece R., Veale D.J., Emery P., Isaacs J.D., Gaston J.S., Crowe J.S. (2001). Detection of Mycobacterium tuberculosis group organisms in human and mouse joInt. tissue by reverse transcriptase PCR: Prevalence in diseased synovial tissue suggests lack of specific association with rheumatoid arthritis. Infect. Immun..

[70] van der Heijden I.M., Wilbrink B., Schouls L.M., van Embden J.D., Breedveld F.C., Tak P.P. (1999). Detection of mycobacteria in joInt. samples from patients with arthritis using a genus-specific polymerase chain reaction and sequence analysis. Rheumatology.

[71] Wu C.H., Jeng K.C., Lan J.L. (1995). Mycobacterium tuberculosis antigen, interleukin 2 and interleukin 2 inhibitor in patients with rheumatoid arthritis. Immunol. Invest..

[72] Erre G.L., Cossu D., Masala S., Mameli G., Cadoni M.L., Serdino S., Longu M.G., Passiu G., Sechi L.A. (2014). Mycobacterium tuberculosis lipoarabinomannan antibodies are associated to rheumatoid arthritis in Sardinian patients. Clin. Rheumatol..

[73] Bahr G.M., Rook G.A., al-Saffar M., Van Embden J., Stanford J.L., Behbehani K. (1988). Antibody levels to mycobacteria in relation to HLA type: Evidence for non-HLA-linked high levels of antibody to the 65 kD heat shock protein of M. bovis in rheumatoid arthritis. Clin. Exp. Immunol..

[74] Tsoulfa G., Rook G.A., Bahr G.M., Sattar M.A., Behbehani K., Young D.B., Mehlert A., Van-Embden J.D., Hay F.C., Isenberg D.A. (1989). Elevated IgG antibody levels to the mycobacterial 65-kDa heat shock protein are characteristic of patients with rheumatoid arthritis. Scand. J. Immunol..

[75] Bo M., Erre G.L., Bach H., Slavin Y.N., Manchia P.A., Passiu G., Sechi L.A. (2019). PtpA and PknG proteins secreted by *Mycobacterium avium* subsp. paratuberculosis are recognized by sera from patients with rheumatoid arthritis: A case-control study. J. Inflamm. Res..

[76] Gaston J.S., Life P.F., Bailey L.C., Bacon P.A. (1989). In vitro responses to a 65-kilodalton mycobacterial protein by synovial T cells from inflammatory arthritis patients. J. Immunol..

[77] Holoshitz J., Koning F., Coligan J.E., De Bruyn J., Strober S. (1989). Isolation of CD4- CD8- mycobacteria-reactive T lymphocyte clones from rheumatoid arthritis synovial fluid. Nature.

[78] Kanagawa H., Niki Y., Kobayashi T., Sato Y., Katsuyama E., Fujie A., Hao W., Miyamoto K., Tando T., Watanabe R. (2015). Mycobacterium tuberculosis promotes arthritis development through Toll-like receptor 2. J. Bone Min. Metab..

[79] Brand D.D., Latham K.A., Rosloniec E.F. (2007). Collagen-induced arthritis. Nat. Protoc..

[80] Hu S., He W., Du X., Yang J., Wen Q., Zhong X.P., Ma L. (2017). IL-17 Production of neutrophils enhances antibacteria ability but promotes arthritis development during mycobacterium tuberculosis infection. EBioMedicine.

[81] Billiau A., Matthys P. (2001). Modes of action of Freund’s adjuvants in experimental models of autoimmune diseases. J. Leukoc. Biol..

[82] Celis L., Vandevyver C., Geusens P., Dequeker J., Raus J., Zhang J. (1997). Clonal expansion of mycobacterial heat-shock protein-reactive T lymphocytes in the synovial fluid and blood of rheumatoid arthritis patients. Arthritis Rheum..

[83] Kogure A., Miyata M., Nishimaki T., Kasukawa R. (1994). Proliferative response of synovial fluid mononuclear cells of patients with rheumatoid arthritis to mycobacterial 65 kDa heat shock protein and its association with HLA-DR+.gamma delta+ T cells. J. Rheumatol..

[84] Wucherpfennig K.W., Strominger J.L. (1995). Molecular mimicry in T cell-mediated autoimmunity: Viral peptides activate human T cell clones specific for myelin basic protein. Cell.

[85] Tsuchiya N., Williams R.C. (1992). Molecular mimicry--hypothesis or reality?. West. J. Med..

[86] Esaguy N., Aguas A.P., van Embden J.D., Silva M.T. (1991). Mycobacteria and human autoimmune disease: Direct evidence of cross-reactivity between human lactoferrin and the 65-kilodalton protein of tubercle and leprosy bacilli. Infect. Immun..

[87] Bizzaro N., Mazzanti G., Tonutti E., Villalta D., Tozzoli R. (2001). Diagnostic accuracy of the anti-citrulline antibody assay for rheumatoid arthritis. Clin. Chem..

[88] Lim M.K., Shim T.S., Sheen D.H., Na D.J., Min S.S., Shim S.C. (2010). Anti-cyclic citrulline peptide antibody in non-tuberculous mycobacteria sera: A negative association. Clin. Rheumatol..

[89] Elkayam O., Segal R., Bendayan D., van Uitert R., Onnekink C., Pruijn G.J. (2010). The anti-cyclic citrullinated peptide response in tuberculosis patients is not citrulline-dependent and sensitive to treatment. Arthritis Res. Ther..

[90] Silva A.F.D., Matos A.N., Lima Á.M.S., Lima E.F., Gaspar A.P., Braga J.A.F., Carvalho E.M. (2006). Valor diagnóstico do anticorpo antipeptídeo citrulinado cíclico na artrite reumatóide. Revista Brasileira de Reumatologia.

[91] Aguas A., Esaguy N., Sunkel C.E., Silva M.T. (1990). Cross-reactivity and sequence homology between the 65-kilodalton mycobacterial heat shock protein and human lactoferrin, transferrin, and DR beta subsets of major histocompatibility complex class II molecules. Infect. Immun..

[92] van Eden W., Holoshitz J., Nevo Z., Frenkel A., Klajman A., Cohen I.R. (1985). Arthritis induced by a T-lymphocyte clone that responds to Mycobacterium tuberculosis and to cartilage proteoglycans. Proc. Natl. Acad. Sci. USA.

[93] van Eden W., Hogervorst E.J., van der Zee R., van Embden J.D., Hensen E.J., Cohen I.R. (1989). The mycobacterial 65 kD heat-shock protein and autoimmune arthritis. Rheumatol. Int..

[94] Dow C.T.M. (2012). paratuberculosis Heat Shock Protein 65 and Human Diseases: Bridging Infection and Autoimmunity. Autoimmune Dis..

[95] Valdez M.M., Clark J.I., Wu G.J., Muchowski P.J. (2002). Functional similarities between the small heat shock proteins Mycobacterium tuberculosis HSP 16.3 and human alphaB-crystallin. Eur. J. BioChem..

[96] Dubaniewicz A., Trzonkowski P., Dubaniewicz-Wybieralska M., Dubaniewicz A., Singh M., Myśliwski A. (2006). Comparative analysis of mycobacterial heat shock proteins-induced apoptosis of peripheral blood mononuclear cells in sarcoidosis and tuberculosis. J. Clin. Immunol..

[97] Hill H.M., Kirshbaum J.D. (1956). Military tuberculosis developing during prolonged cortisone therapy of systemic lupus erythematosus. Ann. Intern. Med..

[98] Ribeiro F.M., Szyper-Kravitz M., Klumb E.M., Lannes G., Ribeiro F.R., Albuquerque E.M., Shoenfeld Y. (2010). Can lupus flaRes. be associated with tuberculosis infection?. Clin. Rev. Allergy Immunol..

[99] van Eden W., Thole J.E., van der Zee R., Noordzij A., van Embden J.D., Hensen E.J., Cohen I.R. (1988). Cloning of the mycobacterial epitope recognized by T lymphocytes in adjuvant arthritis. Nature.

[100] Zügel U., Kaufmann S.H. (1999). Role of heat shock proteins in protection from and pathogenesis of infectious diseases. Clin. Microbiol. Rev..

[101] Dubaniewicz A., Dubaniewicz-Wybieralska M., Sternau A., Zwolska Z., Izycka-Swieszewska E., Augustynowicz-Kopec E., Skokowski J., Singh M., Zimnoch L. (2006). Mycobacterium tuberculosis complex and mycobacterial heat shock proteins in lymph node tissue from patients with pulmonary sarcoidosis. J. Clin. Microbiol..

[102] van Eden W., van der Zee R., Prakken B. (2005). Heat-shock proteins induce T-cell regulation of chronic inflammation. Nat. Rev. Immunol..

[103] Shoda H., Hanata N., Sumitomo S., Okamura T., Fujio K., Yamamoto K. (2016). Immune responses to Mycobacterial heat shock protein 70 accompany self-reactivity to human BiP in rheumatoid arthritis. Sci. Rep..

[104] Chodisetti S.B., Rai P.K., Gowthaman U., Pahari S., Agrewala J.N. (2012). Potential T cell epitopes of Mycobacterium tuberculosis that can instigate molecular mimicry against host: Implications in autoimmune pathogenesis. BMC Immunol..

[105] Gutlapalli V.R., Sykam A., Nayarisseri A., Suneetha S., Suneetha L.M. (2015). Insights from the predicted epitope similarity between Mycobacterium tuberculosis virulent factors and its human homologs. Bioinformation.

[106] Mustafa A.S., Al-Attiyah R., Hanif S.N., Shaban F.A. (2008). Efficient testing of large pools of Mycobacterium tuberculosis RD1 peptides and identification of major antigens and immunodominant peptides recognized by human Th1 cells. Clin. Vaccine Immunol..

[107] Mustafa A.S. (2010). In silico binding predictions for identification of HLA-DR-promiscuous regions and epitopes of Mycobacterium tuberculosis protein MPT64 (Rv1980c) and their recognition by human Th1 cells. Med. Princ. Pract..

[108] Gowthaman U., Agrewala J.N. (2009). In silico methods for predicting T-cell epitopes: Dr Jekyll or Mr Hyde?. Expert Rev. Proteom..

[109] Ates O., Dalyan L., Müsellim B., Hatemi G., Türker H., Ongen G., Hamuryudan V., Topal-Sarikaya A. (2009). NRAMP1 (SLC11A1) gene polymorphisms that correlate with autoimmune versus infectious disease susceptibility in tuberculosis and rheumatoid arthritis. Int. J. Immunogenet..

[110] Sechi L.A., Gazouli M., Sieswerda L.E., Molicotti P., Ahmed N., Ikonomopoulos J., Scanu A.M., Paccagnini D., Zanetti S. (2006). Relationship between Crohn’s disease, infection with Mycobacterium avium subspecies paratuberculosis and SLC11A1 gene polymorphisms in Sardinian patients. World J. Gastroenterol..

[111] Paccagnini D., Sieswerda L., Rosu V., Masala S., Pacifico A., Gazouli M., Ikonomopoulos J., Ahmed N., Zanetti S., Sechi L.A. (2009). Linking chronic infection and autoimmune diseases: Mycobacterium avium subspecies paratuberculosis, SLC11A1 polymorphisms and type-1 diabetes mellitus. PLoS ONE.

[112] Sharp R.C., Beg S.A., Naser S.A. (2018). Polymorphisms in protein tyrosine phosphatase non-receptor type 2 and 22 (PTPN2/22) are linked to Hyper-Proliferative T-Cells and susceptibility to mycobacteria in rheumatoid arthritis. Front. Cell. Infect. Microbiol..

[113] Wyllie S., Seu P., Goss J.A. (2002). The natural resistance-associated macrophage protein 1 Slc11a1 (formerly Nramp1) and iron metabolism in macrophages. Microbes Infect..

[114] Hackam D.J., Rotstein O.D., Zhang W., Gruenheid S., Gros P., Grinstein S. (1998). Host resistance to intracellular infection: Mutation of natural resistance-associated macrophage protein 1 (Nramp1) impairs phagosomal acidification. J. Exp. Med..

[115] Yang Y.S., Kim S.J., Kim J.W., Koh E.M. (2000). NRAMP1 gene polymorphisms in patients with rheumatoid arthritis in Koreans. J. Korean Med. Sci..

[116] Kotze M.J., de Villiers J.N., Rooney R.N., Grobbelaar J.J., Mansvelt E.P., Bouwens C.S., Carr J., Stander I., du Plessis L. (2001). Analysis of the NRAMP1 gene implicated in iron transport: Association with multiple sclerosis and age effects. Blood Cells Mol. Dis..

[117] Gazouli M., Sechi L., Paccagnini D., Sotgiu S., Arru G., Nasioulas G., Vassilopoulos D. (2008). NRAMP1 polymorphism and viral factors in Sardinian multiple sclerosis patients. Can. J. Neurol. Sci..

[118] Kotlowski R., Bernstein C.N., Silverberg M.S., Krause D.O. (2008). Population-based case-control study of alpha 1-antitrypsin and SLC11A1 in Crohn’s disease and ulcerative colitis. Inflamm. Bowel Dis..

[119] Takahashi K., Satoh J., Kojima Y., Negoro K., Hirai M., Hinokio Y., Kinouchi Y., Suzuki S., Matsuura N., Shimosegawa T. (2004). Promoter polymorphism of SLC11A1 (formerly NRAMP1) confers susceptibility to autoimmune type 1 diabetes mellitus in Japanese. Tissue Antigens.

[120] Qasem A., Ramesh S., Naser S.A. (2019). Genetic polymorphisms in tumour necrosis factor receptors (TNFRSF1A/1B) illustrate differential treatment response to TNFα inhibitors in patients with Crohn’s disease. BMJ Open Gastroenterol..

[121] Matsui T., Ohsumi K., Ozawa N., Shimada K., Sumitomo S., Shimane K., Kawakami M., Nakayama H., Sugii S., Ozawa Y. (2006). CD64 on neutrophils is a sensitive and specific marker for detection of infection in patients with rheumatoid arthritis. J. Rheumatol..

[122] Caporali R., Caprioli M., Bobbio-Pallavicini F., Montecucco C. (2008). DMARDS and infections in rheumatoid arthritis. AutoImmun. Rev..

[123] Solovic I., Sester M., Gomez-Reino J.J., Rieder H.L., Ehlers S., Milburn H.J., Kampmann B., Hellmich B., Groves R., Schreiber S. (2010). The risk of tuberculosis related to tumour necrosis factor antagonist therapies: A TBNET consensus statement. Eur. Respir. J..

[124] Askling J., Fored C.M., Brandt L., Baecklund E., Bertilsson L., Cöster L., Geborek P., Jacobsson L.T., Lindblad S., Lysholm J. (2005). Risk and case characteristics of tuberculosis in rheumatoid arthritis associated with tumor necrosis factor antagonists in Sweden. Arthritis Rheum..

[125] Ingraham N.E., Schneider B., Alpern J.D. (2017). Prosthetic JoInt. Infection due to Mycobacterium avium-intracellulare in a Patient with Rheumatoid Arthritis: A Case Report and Review of the Literature. Case Rep. Infect. Dis..

[126] Iwata K., Oka S., Tsuno H., Furukawa H., Shimada K., Hashimoto A., Komiya A., Tsuchiya N., Katayama M., Tohma S. (2018). Biomarker for nontuberculous mycobacterial pulmonary disease in patients with rheumatoid arthritis: Anti-glycopeptidolipid core antigen immunoglobulin A antibodies. Mod. Rheumatol..

[127] Schubert N., Schill T., Plüß M., Korsten P. (2020). Flare or foe?—Mycobacterium marinum infection mimicking rheumatoid arthritis tenosynovitis: Case report and literature review. BMC Rheumatol..

[128] Chen H.W., Lai C.C., Tan C.K. (2009). Arthritis caused by Mycobacterium terrae in a patient with rheumatoid arthritis. Int. J. Infect Dis..

[129] Lam A., Toma W., Schlesinger N. (2006). Mycobacterium marinum arthritis mimicking rheumatoid arthritis. J. Rheumatol..

[130] DeMerieux P., Keystone E.C., Hutcheon M., Laskin C. (1980). Polyarthritis due to Mycobacterium kansasii in a patient with rheumatoid arthritis. Ann. Rheum. Dis..

[131] Dos Santos Sobrín R., Pérez Gómez N., Vilas A.S., Suárez M.P., Pampín E.P., Antúnez López J.R., Mera Varela A. (2020). Infection by Mycobacterium chelonae at the site of administration of sarilumab for rheumatoid arthritis. Rheumatology.

[132] Dutertre M., Delobel P., Marchou B., Boyer J.F., Mougari F., Martin-Blondel G. (2019). Olecranon bursitis secondary to Mycobacterium europaeum infection in a patient receiving immunosuppressive drugs for rheumatoid arthritis. Med. Et Mal. Infect..

[133] Benedek T.G. (2006). The history of bacteriologic concepts of rheumatic fever and rheumatoid arthritis. Semin Arthritis Rheum..

[134] Martinez-Martinez R.E., Abud-Mendoza C., Patiño-Marin N., Rizo-Rodríguez J.C., Little J.W., Loyola-Rodríguez J.P. (2009). Detection of periodontal bacterial DNA in serum and synovial fluid in refractory rheumatoid arthritis patients. J. Clin. Periodontol..

[135] Totaro M.C., Cattani P., Ria F., Tolusso B., Gremese E., Fedele A.L., D’Onghia S., Marchetti S., Sante G.D., Canestri S. (2013). Porphyromonas gingivalis and the pathogenesis of rheumatoid arthritis: Analysis of various compartments including the synovial tissue. Arthritis Res. Ther..

[136] Kawahito Y., Ichinose S., Sano H., Tsubouchi Y., Kohno M., Yoshikawa T., Tokunaga D., Hojo T., Harasawa R., Nakano T. (2008). Mycoplasma fermentans glycolipid-antigen as a pathogen of rheumatoid arthritis. BioChem. Biophys. Res. Commun..

[137] Schaeverbeke T., Renaudin H., Clerc M., Lequen L., Vernhes J.P., De Barbeyrac B., Bannwarth B., Bébéar C., Dehais J. (1997). Systematic detection of mycoplasmas by culture and polymerase chain reaction (PCR) proceduRes. in 209 synovial fluid samples. Rheumatology.

[138] Hoffman R.W., O’Sullivan F.X., Schafermeyer K.R., Moore T.L., Roussell D., Watson-McKown R., Kim M.F., Wise K.S. (1997). Mycoplasma infection and rheumatoid arthritis: Analysis of their relationship using immunoblotting and an ultrasensitive polymerase chain reaction detection method. Arthritis Rheum..

[139] Wilkinson N.Z., Kingsley G.H., Jones H.W., Sieper J., Braun J., Ward M.E. (1999). The detection of DNA from a range of bacterial species in the joints of patients with a variety of arthritides using a nested, broad-range polymerase chain reaction. Rheumatology.

[140] van der Heijden I.M., Wilbrink B., Tchetverikov I., Schrijver I.A., Schouls L.M., Hazenberg M.P., Breedveld F.C., Tak P.P. (2000). Presence of bacterial DNA and bacterial peptidoglycans in joints of patients with rheumatoid arthritis and other arthritides. Arthritis Rheum..

[141] Saal J.G., Steidle M., Einsele H., Müller C.A., Fritz P., Zacher J. (1992). Persistence of B19 parvovirus in synovial membranes of patients with rheumatoid arthritis. Rheumatol. Int..

[142] Jobanputra P., Davidson F., Graham S., O’Neill H., Simmonds P., Yap P.L. (1995). High frequency of parvovirus B19 in patients tested for rheumatoid factor. BMJ.

[143] Takeda T., Mizugaki Y., Matsubara L., Imai S., Koike T., Takada K. (2000). Lytic Epstein-Barr virus infection in the synovial tissue of patients with rheumatoid arthritis. Arthritis Rheum..

[144] Mehraein Y., Lennerz C., Ehlhardt S., Remberger K., Ojak A., Zang K.D. (2004). Latent Epstein-Barr virus (EBV) infection and cytomegalovirus (CMV) infection in synovial tissue of autoimmune chronic arthritis determined by RNA- and DNA-in situ hybridization. Mod. Pathol..

[145] Sorgato C.C., Lins E.S.M., Leão J.C., Vasconcelos L.R., Melo T.R., Duarte A.L., Gueiros L.A. (2020). EBV and CMV Viral Load in Rheumatoid Arthritis and Their Role Associated Sjögren’s Syndrome. J. Oral. Pathol. Med..

[146] Kuusela E., Kouri V.P., Olkkonen J., Koivuniemi R., Äyräväinen L., Rajamäki K., Valleala H., Nordström D., Leirisalo-Repo M., Ainola M. (2018). Serum Epstein-Barr virus DNA, detected by droplet digital PCR, correlates with disease activity in patients with rheumatoid arthritis. Clin. Exp. Rheumatol..

[147] Erre G.L., Mameli G., Cossu D., Muzzeddu B., Piras C., Paccagnini D., Passiu G., Sechi L.A. (2015). Increased Epstein-Barr Virus DNA Load and Antibodies Against EBNA1 and EA in Sardinian Patients with Rheumatoid Arthritis. Viral. Immunol..

[148] Mikuls T.R., Payne J.B., Reinhardt R.A., Thiele G.M., Maziarz E., Cannella A.C., Holers V.M., Kuhn K.A., O’Dell J.R. (2009). Antibody responses to Porphyromonas gingivalis (P. gingivalis) in subjects with rheumatoid arthritis and periodontitis. Int. Immunopharmacol..

[149] Ogrendik M., Kokino S., Ozdemir F., Bird P.S., Hamlet S. (2005). Serum antibodies to oral anaerobic bacteria in patients with rheumatoid arthritis. MedGenMed.

[150] Quirke A.M., Lugli E.B., Wegner N., Hamilton B.C., Charles P., Chowdhury M., Ytterberg A.J., Zubarev R.A., Potempa J., Culshaw S. (2014). Heightened immune response to autocitrullinated Porphyromonas gingivalis peptidylarginine deiminase: A potential mechanism for breaching immunologic tolerance in rheumatoid arthritis. Ann. Rheum. Dis..

[151] Klatt T., Ouyang Q., Flad T., Koetter I., Bühring H.J., Kalbacher H., Pawelec G., Müller C.A. (2005). Expansion of peripheral CD8+ CD28- T cells in response to Epstein-Barr virus in patients with rheumatoid arthritis. J. Rheumatol..

[152] Rickinson A.B., Moss D.J. (1997). Human cytotoxic T lymphocyte responses to Epstein-Barr virus infection. Annu. Rev. Immunol..

[153] Scotet E., David-Ameline J., Peyrat M.A., Moreau-Aubry A., Pinczon D., Lim A., Even J., Semana G., Berthelot J.M., Breathnach R. (1996). T cell response to Epstein-Barr virus transactivators in chronic rheumatoid arthritis. J. Exp. Med..

[154] Lünemann J.D., Frey O., Eidner T., Baier M., Roberts S., Sashihara J., Volkmer R., Cohen J.I., Hein G., Kamradt T. (2008). Increased frequency of EBV-specific effector memory CD8+ T cells correlates with higher viral load in rheumatoid arthritis. J. Immunol..

[155] Trier N.H., Holm B.E., Heiden J., Slot O., Locht H., Lindegaard H., Svendsen A., Nielsen C.T., Jacobsen S., Theander E. (2018). Antibodies to a strain-specific citrullinated Epstein-Barr virus peptide diagnoses rheumatoid arthritis. Sci. Rep..

[156] Sternbæk L., Draborg A.H., Østerlund M.T., Iversen L.V., Troelsen L., Theander E., Nielsen C.T., Jacobsen S., Houen G. (2019). Increased antibody levels to stage-specific Epstein-Barr virus antigens in systemic autoimmune diseases reveal a common pathology. Scand. J. Clin. Lab. Investig..

[157] Motokawa S., Hasunuma T., Tajima K., Krieg A.M., Ito S., Iwasaki K., Nishioka K. (1996). High prevalence of arthropathy in HTLV-I carriers on a Japanese island. Ann. Rheum. Dis..

[158] da Rocha Sobrinho H.M., Jarach R., da Silva N.A., Shio M.T., Jancar S., Timenetsky J., Oliveira M.A., Dorta M.L., Ribeiro-Dias F. (2011). Mycoplasmal lipid-associated membrane proteins and Mycoplasma arthritidis mitogen recognition by serum antibodies from patients with rheumatoid arthritis. Rheumatol. Int..

[159] Sawitzke A., Joyner D., Knudtson K., Mu H.H., Cole B. (2000). Anti-MAM antibodies in rheumatic disease: Evidence for a MAM-like superantigen in rheumatoid arthritis?. J. Rheumatol..

[160] Tzang B.S., Tsai C.C., Tsay G.J., Wang M., Sun Y.S., Hsu T.C. (2009). Anti-human parvovirus B19 nonstructural protein antibodies in patients with rheumatoid arthritis. Clin. Chim. Acta.

[161] Shi J., Sun X., Zhao Y., Zhao J., Li Z. (2008). Prevalence and significance of antibodies to citrullinated human papilloma virus-47 E2345-362 in rheumatoid arthritis. J. AutoImmun..

[162] Mameli G., Erre G.L., Caggiu E., Mura S., Cossu D., Bo M., Cadoni M.L., Piras A., Mundula N., Colombo E. (2017). Identification of a HERV-K env surface peptide highly recognized in Rheumatoid Arthritis (RA) patients: A cross-sectional case-control study. Clin. Exp. Immunol..

[163] Chukkapalli S., Rivera-Kweh M., Gehlot P., Velsko I., Bhattacharyya I., Calise S.J., Satoh M., Chan E.K., Holoshitz J., Kesavalu L. (2016). Periodontal bacterial colonization in synovial tissues exacerbates collagen-induced arthritis in B10.RIII mice. Arthritis Res. Ther..

[164] Jung H., Jung S.M., Rim Y.A., Park N., Nam Y., Lee J., Park S.-H., Ju J.H. (2017). Arthritic role of Porphyromonas gingivalis in collagen-induced arthritis mice. PLoS ONE.

[165] Yamakawa M., Ouhara K., Kajiya M., Munenaga S., Kittaka M., Yamasaki S., Takeda K., Takeshita K., Mizuno N., Fujita T. (2016). Porphyromonas gingivalis infection exacerbates the onset of rheumatoid arthritis in SKG mice. Clin. Exp. Immunol..

[166] Bartold P.M., Marino V., Cantley M., Haynes D.R. (2010). Effect of Porphyromonas gingivalis-induced inflammation on the development of rheumatoid arthritis. J. Clin. Periodontol..

[167] Cole B.C., Golightly-Rowland L., Ward J.R. (1976). Arthritis of mice induced by Mycoplasma arthritidis. Humoral antibody and lymphocyte responses of CBA mice. Ann. Rheum. Dis..

[168] Kuwana Y., Takei M., Yajima M., Imadome K.-I., Inomata H., Shiozaki M., Ikumi N., Nozaki T., Shiraiwa H., Kitamura N. (2011). Epstein-Barr Virus Induces Erosive Arthritis in Humanized Mice. PLoS ONE.

[169] Fujiwara S., Matsuda G., Imadome K.-I. (2013). Humanized mouse models of epstein-barr virus infection and associated diseases. Pathogens.

[170] Röhner E., Detert J., Kolar P., Hocke A., N’Guessan P., Matziolis G., Kanitz V., Bernimoulin J.P., Kielbassa A., Burmester G.R. (2010). Induced apoptosis of chondrocytes by Porphyromonas gingivalis as a possible pathway for cartilage loss in rheumatoid arthritis. Calcif. Tissue Int..

[171] Lee J.Y., Choi I.A., Kim J.H., Kim K.H., Lee E.Y., Lee E.B., Lee Y.M., Song Y.W. (2015). Association between anti-Porphyromonas gingivalis or anti-α-enolase antibody and severity of periodontitis or rheumatoid arthritis (RA) disease activity in RA. BMC Musculoskelet. Disord..

[172] Rojas M., Restrepo-Jiménez P., Monsalve D.M., Pacheco Y., Acosta-Ampudia Y., Ramírez-Santana C., Leung P.S.C., Ansari A.A., Gershwin M.E., Anaya J.M. (2018). Molecular mimicry and autoimmunity. J. AutoImmun..

[173] Lundberg K., Kinloch A., Fisher B.A., Wegner N., Wait R., Charles P., Mikuls T.R., Venables P.J. (2008). Antibodies to citrullinated alpha-enolase peptide 1 are specific for rheumatoid arthritis and cross-react with bacterial enolase. Arthritis Rheum..

[174] Bo M., Erre G.L., Niegowska M., Piras M., Taras L., Longu M.G., Passiu G., Sechi L.A. (2018). Interferon regulatory factor 5 is a potential target of autoimmune response triggered by Epstein-barr virus and Mycobacterium avium subsp. paratuberculosis in rheumatoid arthritis: Investigating a mechanism of molecular mimicry. Clin. Exp. Rheumatol..

[175] Ebringer A., Rashid T., Wilson C. (2010). Rheumatoid arthritis, Proteus, anti-CCP antibodies and Karl Popper. AutoImmun. Rev..

[176] Ebringer A., Rashid T. (2006). Rheumatoid arthritis is an autoimmune disease triggered by Proteus urinary tract infection. Clin. Dev. Immunol..

[177] Wilson C., Ebringer A., Ahmadi K., Wrigglesworth J., Tiwana H., Fielder M., Binder A., Ettelaie C., Cunningham P., Joannou C. (1995). Shared amino acid sequences between major histocompatibility complex class II glycoproteins, type XI collagen and Proteus mirabilis in rheumatoid arthritis. Ann. Rheum. Dis..

[178] Ebringer A., Rashid T. (2014). Rheumatoid arthritis is caused by a Proteus urinary tract infection. Apmis.

[179] Tiwana H., Wilson C., Alvarez A., Abuknesha R., Bansal S., Ebringer A. (1999). Cross-reactivity between the rheumatoid arthritis-associated motif EQKRAA and structurally related sequences found in Proteus mirabilis. Infect. Immun..

[180] Wilson C., Tiwana H., Ebringer A. (2000). Molecular mimicry between HLA-DR alleles associated with rheumatoid arthritis and Proteus mirabilis as the Aetiological basis for autoimmunity. Microbes Infect..

[181] Hou Y., Lin H., Zhu L., Liu Z., Hu F., Shi J., Yang T., Shi X., Zhu M., Godley B.F. (2013). Lipopolysaccharide increases the incidence of collagen-induced arthritis in mice through induction of protease HTRA-1 expression. Arthritis Rheum..

[182] Lorenz W., Buhrmann C., Mobasheri A., Lueders C., Shakibaei M. (2013). Bacterial lipopolysaccharides form procollagen-endotoxin complexes that trigger cartilage inflammation and degeneration: Implications for the development of rheumatoid arthritis. Arthritis Res. Ther..

[183] Nakayama M., Niki Y., Kawasaki T., Takeda Y., Horiuchi K., Sasaki A., Okada Y., Umezawa K., Ikegami H., Toyama Y. (2012). Enhanced susceptibility to lipopolysaccharide-induced arthritis and endotoxin shock in interleukin-32 alpha transgenic mice through induction of tumor necrosis factor alpha. Arthritis Res. Ther..

[184] Yücel G., Zhao Z., El-Battrawy I., Lan H., Lang S., Li X., Buljubasic F., Zimmermann W.-H., Cyganek L., Utikal J. (2017). Lipopolysaccharides induced inflammatory responses and electrophysiological dysfunctions in human-induced pluripotent stem cell derived cardiomyocytes. Sci. Rep..

[185] Frost R.A., Nystrom G.J., Lang C.H. (2002). Lipopolysaccharide regulates proinflammatory cytokine expression in mouse myoblasts and skeletal muscle. Am. J. Physiol. Regul. Integr. Comp. Physiol..

[186] Barksby H.E., Nile C.J., Jaedicke K.M., Taylor J.J., Preshaw P.M. (2009). Differential expression of immunoregulatory genes in monocytes in response to Porphyromonas gingivalis and Escherichia coli lipopolysaccharide. Clin. Exp. Immunol..

[187] Nile C.J., Barksby E., Jitprasertwong P., Preshaw P.M., Taylor J.J. (2010). Expression and regulation of interleukin-33 in human monocytes. Immunology.

[188] Burns E., Bachrach G., Shapira L., Nussbaum G. (2006). Cutting Edge: TLR2 is required for the innate response to Porphyromonas gingivalis: Activation leads to bacterial persistence and TLR2 deficiency attenuates induced alveolar bone resorption. J. Immunol..

[189] Lin J., Bi L., Yu X., Kawai T., Taubman M.A., Shen B., Han X. (2014). Porphyromonas gingivalis exacerbates ligature-induced, RANKL-dependent alveolar bone resorption via differential regulation of Toll-like receptor 2 (TLR2) and TLR4. Infect. Immun..

[190] Yang S., Tamai R., Akashi S., Takeuchi O., Akira S., Sugawara S., Takada H. (2001). Synergistic effect of muramyldipeptide with lipopolysaccharide or lipoteichoic acid to induce inflammatory cytokines in human monocytic cells in culture. Infect. Immun..

[191] Yang S., Takahashi N., Yamashita T., Sato N., Takahashi M., Mogi M., Uematsu T., Kobayashi Y., Nakamichi Y., Takeda K. (2005). Muramyl Dipeptide Enhances Osteoclast Formation Induced by Lipopolysaccharide, IL-1α, and TNF-α through Nucleotide-Binding Oligomerization Domain 2-Mediated Signaling in Osteoblasts. J. Immunol..

[192] Shehab M., Sherri N., Hussein H., Salloum N., Rahal E.A. (2019). Endosomal Toll-Like Receptors Mediate Enhancement of Interleukin-17A Production Triggered by Epstein-Barr Virus DNA in Mice. J. Virol..

[193] Salloum N., Hussein H.M., Jammaz R., Jiche S., Uthman I.W., Abdelnoor A.M., Rahal E.A. (2018). Epstein-Barr virus DNA modulates regulatory T-cell programming in addition to enhancing interleukin-17A production via Toll-like receptor 9. PLoS ONE.

[194] Hsiao F.C., Lin M., Tai A., Chen G., Huber B.T. (2006). Cutting edge: Epstein-Barr virus transactivates the HERV-K18 superantigen by docking to the human complement receptor 2 (CD21) on primary B cells. J. Immunol..

[195] Ford D.K., da Roza D.M., Schulzer M., Reid G.D., Denegri J.F. (1987). Persistent synovial lymphocyte responses to cytomegalovirus antigen in some patients with rheumatoid arthritis. Arthritis Rheum..

[196] Kinloch A.J., Alzabin S., Brintnell W., Wilson E., Barra L., Wegner N., Bell D.A., Cairns E., Venables P.J. (2011). Immunization with Porphyromonas gingivalis enolase induces autoimmunity to mammalian α-enolase and arthritis in DR4-IE-transgenic mice. Arthritis Rheum..

[197] Wegner N., Wait R., Sroka A., Eick S., Nguyen K.A., Lundberg K., Kinloch A., Culshaw S., Potempa J., Venables P.J. (2010). Peptidylarginine deiminase from Porphyromonas gingivalis citrullinates human fibrinogen and α-enolase: Implications for autoimmunity in rheumatoid arthritis. Arthritis Rheum..

[198] Maresz K.J., Hellvard A., Sroka A., Adamowicz K., Bielecka E., Koziel J., Gawron K., Mizgalska D., Marcinska K.A., Benedyk M. (2013). Porphyromonas gingivalis facilitates the development and progression of destructive arthritis through its unique bacterial peptidylarginine deiminase (PAD). PloS Pathog..

[199] Courbon G., Rinaudo-Gaujous M., Blasco-Baque V., Auger I., Caire R., Mijola L., Vico L., Paul S., Marotte H. (2019). Porphyromonas gingivalis experimentally induces periodontis and an anti-CCP2-associated arthritis in the rat. Ann. Rheum. Dis..

